# Hypoxia enhances human myoblast differentiation: involvement of HIF1α and impact of DUX4, the FSHD causal gene

**DOI:** 10.1186/s13395-023-00330-2

**Published:** 2023-12-16

**Authors:** Thuy-Hang Nguyen, Lise Paprzycki, Alexandre Legrand, Anne-Emilie Declèves, Philipp Heher, Maelle Limpens, Alexandra Belayew, Christopher R. S. Banerji, Peter S. Zammit, Alexandra Tassin

**Affiliations:** 1 Laboratory of Respiratory Physiology, Pathophysiology and Rehabilitation, Research Institute for Health Sciences and Technology, University of Mons, Mons, 7000 Belgium; 2 Department of Metabolic and Molecular Biochemistry, Research Institute for Health Sciences and Technology, University of Mons, Mons, 7000 Belgium; 3 Randall Centre for Cell and Molecular Biophysics, King’s College London, Guy’s Campus, London, SE1 1UL UK; 4 The Alan Turing Institute, British Library, 96 Euston Rd, London, UK

## Abstract

**Background:**

Hypoxia is known to modify skeletal muscle biological functions and muscle regeneration. However, the mechanisms underlying the effects of hypoxia on human myoblast differentiation remain unclear. The hypoxic response pathway is of particular interest in patients with hereditary muscular dystrophies since many present respiratory impairment and muscle regeneration defects. For example, an altered hypoxia response characterizes the muscles of patients with facioscapulohumeral dystrophy (FSHD).

**Methods:**

We examined the impact of hypoxia on the differentiation of human immortalized myoblasts (LHCN-M2) cultured in normoxia (PO_2_: 21%) or hypoxia (PO_2_: 1%). Cells were grown in proliferation (myoblasts) or differentiation medium for 2 (myocytes) or 4 days (myotubes). We evaluated proliferation rate by EdU incorporation, used myogenin-positive nuclei as a differentiation marker for myocytes, and determined the fusion index and myosin heavy chain-positive area in myotubes. The contribution of HIF1α was studied by gain (CoCl_2_) and loss (siRNAs) of function experiments. We further examined hypoxia in LHCN-M2-iDUX4 myoblasts with inducible expression of DUX4, the transcription factor underlying FSHD pathology.

**Results:**

We found that the hypoxic response did not impact myoblast proliferation but activated precocious myogenic differentiation and that HIF1α was critical for this process. Hypoxia also enhanced the late differentiation of human myocytes, but in an HIF1α-independent manner. Interestingly, the impact of hypoxia on muscle cell proliferation was influenced by dexamethasone. In the FSHD pathological context, DUX4 suppressed HIF1α-mediated precocious muscle differentiation.

**Conclusion:**

Hypoxia stimulates myogenic differentiation in healthy myoblasts, with HIF1α-dependent early steps. In FSHD, DUX4-HIF1α interplay indicates a novel mechanism by which DUX4 could interfere with HIF1α function in the myogenic program and therefore with FSHD muscle performance and regeneration.

**Supplementary Information:**

The online version contains supplementary material available at 10.1186/s13395-023-00330-2.

## Introduction

An imbalance between the O_2_ supply and its requirements at the tissue level leads to a condition termed hypoxia. At the cellular level, response to hypoxia is activated through the stabilization of a key effector, the Hypoxia Inducible Factor 1α (HIF1α). Under hypoxia, HIF1α is stabilized allowing its translocation into the nucleus where it activates the transcription of more than a hundred target genes through binding to their Hypoxia Response Element (HRE) [1].

Skeletal muscle has a very efficient regeneration capacity which is part of its high plasticity. Upon injury, skeletal muscle can initiate a rapid and extensive repair process to prevent the loss of muscle mass mainly through activation of satellite cells (SC), resident quiescent adult muscle stem cells that express the PAX7 transcription factor [2]. After a muscle injury, SC are activated and proliferate to generate myoblast progeny. Myoblasts later differentiate into myocytes to finally fuse either to form new multinucleated myotubes or to repair the damaged myofibers. Activation of myogenic transcription factors (MYF5, MYOD, Myogenin, and MRF4) controls the stages of SC activation and myogenic differentiation [3].

The effects of hypoxia on myogenesis and its influence on myoblast differentiation into myotubes in vitro and on muscle regeneration in vivo have been widely studied but remain unclear [4]. For instance, *Hif1a* deletion in Pax7-positive SC in a mouse model was found to cause increased density of myofibers with central nuclei (day 7 post-injury) and enhanced hypertrophic growth (day 14) in regenerated muscle fibers [5]. Another study in vitro showed that Hif-1α silencing in C2C12 murine muscle cells significantly altered the differentiation process [6]. Similarly, mimicking hypoxia during skeletal muscle regeneration in rats by using dimethyloxalylglycine (DMOG) induced a defect in the activation of *Myf-5* and *Myogenin* [7] while moderate hypoxia promoted C2C12 cell differentiation [8]. To summarize, the effects of hypoxia on myogenic differentiation are not completely understood, and discrepancies between studies likely relate to the different experimental parameters such as the duration, depth, and type of hypoxia (chemical, normo/hypobaric) as well as the muscle model (e.g., immortal cell line *vs*. primary cells), culture media, species (e.g., mouse *vs*. human), and experimental set up (in vitro or in vivo). Also, most data have been collected on mouse muscle cells, with only rare experiments focused on the effects of hypoxia on human myoblasts [9, 10].

Recent reports have highlighted HIF1α as a regulator of myogenesis and SC function. Indeed, HIF1α is involved in mechanisms governing the quiescence of SC in their hypoxic niche [11]. Moreover, HIF1α has a pro-angiogenic role in skeletal muscle, notably through induction of *VEGF* expression, promoting blood capillary development [12], particularly in muscle subjected to exercise training [13, 14]. Accordingly, prolyl hydroxylase (PHD) 2 deficiency and the subsequent Hif1α accumulation in mice enhance muscle regeneration after a myotrauma, with accelerated macrophage recruitment to the injured area [15]. Taken together, these studies suggest that HIF1α is necessary for myogenic differentiation and maintenance under physiological conditions.

The role of HIF1α in pathological conditions has also been highlighted, notably in the context of muscular dystrophies. Indeed, a significant subgroup of patients experience respiratory impairments and subsequently hypoxemia that leads to HIF1α activation. Additional mechanisms can modify HIF1α activity and hypoxia-response pathway in skeletal muscles of patients with muscular dystrophy, notably blood vessel alterations and the genetic defect itself (as we reviewed in [4]).

Intriguingly, the hereditary, progressive myopathy facioscapulohumeral muscular dystrophy (FSHD) is associated with an altered hypoxia response pathway [16, 17]. This association was deduced from a meta-analysis of transcriptome profiling datasets obtained from FSHD muscle biopsies that generated a unified molecular map of FSHD-associated signaling networks [16]. The disturbed hypoxic response therefore constitutes a typical characteristic of FSHD muscle, suggesting a causal link with FSHD etiologic mechanisms per se, rather than a secondary consequence in a particular patient subgroup. The molecular mechanism of FSHD is complex and involves both genetic and epigenetic components leading to the activation in skeletal muscle of *DUX4*, a gene normally mostly expressed in germline and early embryogenesis (reviewed in [18]). *DUX4* encodes a potent transcription activator [19–24] affecting multiple pathways (I) by modulating direct target genes, (II) by dysregulating post-transcriptional processes [25], and (III) by repressing the PAX7 target gene signature [26]. However, DUX4 pathomechanisms are not completely understood. Intriguingly, PAX7 target gene repression was associated with the induction of hypoxia-response genes [17]. HIF1α-signaling is also one of the over-represented pathways among FSHD dysregulated genes [27]. Finally, a link between DUX4 and HIF1α was reported from a genome-wide CRISPR-Cas9 screen to identify genes whose loss-of-function allowed muscle cell survival when DUX4 was expressed: the cellular hypoxia response pathway was identified as the main driver of DUX4-induced cell death [28]. In our recent study, we could confirm the DUX4 and HIF1α link and found that it differed according to the stage of myogenic differentiation. Indeed DUX4 downregulated the HIF1α pathway in myoblasts and activated it in myotubes. Interestingly, we showed that this axis was conserved between human and mouse muscle (manuscript under review in IJMS journal, available in pre-print) [29].

Interestingly, myogenic differentiation, a process known to be regulated by HIF1α, is typically altered in FSHD [23, 26, 30, 31]. FSHD is characterized by progressive rostro-caudal muscle weakness and wasting, associated with low levels of myofiber regeneration [32] which suggests poor SC function. FSHD was recently classified as a secondary satellite cell-opathy, a new classification regrouping muscle disorders in which the causal mutation not only negatively affects muscle fibres but also causes SC dysfunction, affecting muscle regeneration and therefore contributing to pathology [33, 34]. Moreover, FSHD myotubes display morphological features of aberrant differentiation leading to two major phenotypes: (I) myotubes with a thin, elongated morphology described as hypotrophic (also named “atrophic”); (II) myotubes displaying clusters of myonuclei and dysregulation of the microtubule network, considered “disorganized” [30, 31]. We therefore hypothesize that DUX4-induced HIF1α pathway mis-regulation could participate in FSHD-associated defects in adult myogenesis.

Considering (I) the role of HIF1α in myogenesis and muscle regeneration, (II) the dysregulation of HIF1α in FSHD muscles, and (III) that myogenic differentiation defects are a major characteristic of FSHD muscle cells [23, 26, 30, 31], we hypothesized that an inadequate HIF1α activation could participate in FSHD pathophysiology. We first investigated whether sustained HIF1α activation could impact human muscle cell differentiation in vitro. Since DUX4 was reported to alter the HIF1α pathway, we also investigated the impact of DUX4 expression on the hypoxic response of myoblasts during differentiation.

## Results

### Hypoxia enhances early and late myogenic differentiation of human myoblasts

**Fig. 1:**
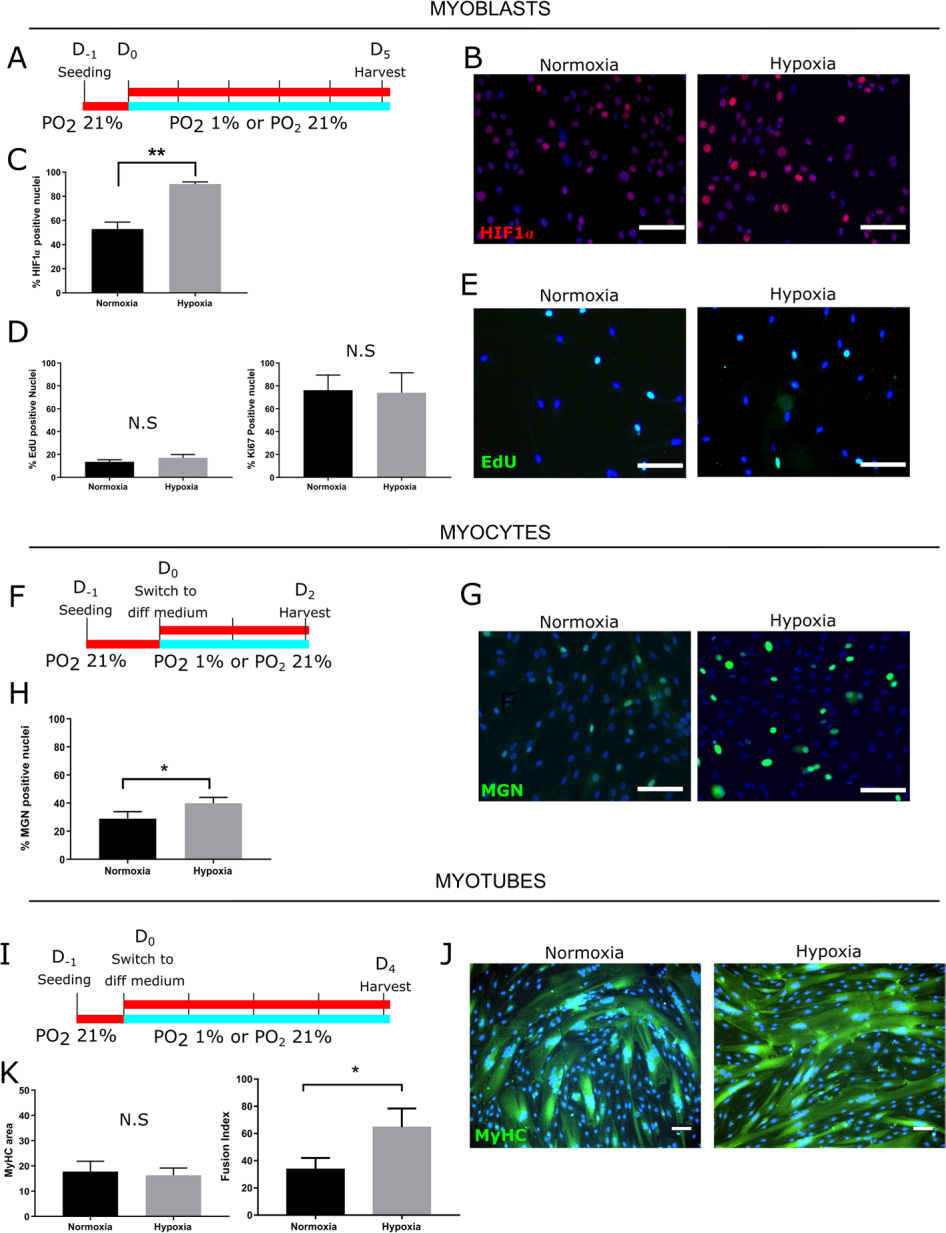
Hypoxia enhances early and late differentiation of human myoblasts. LHCN-M2 myoblasts were seeded in a 6-well plate in standard conditions and 24h later exposed to hypoxia (PO_2_: 1%—blue line) or maintained in standard conditions (PO_2_: 21%—red line). After exposure, cells were fixed, proteins of interest were immunolabelled, and positive nuclei normalized to the total number of nuclei (DAPI; blue staining). Representative fields are shown. Scale bar: 100 μm. Experiments were performed on 3 independent cultures (each in triplicate) and mean ± SEM are represented and compared (*T*-test). *Upper panel: Myoblasts*. **A** 250,000 myoblasts were seeded per well. After 24h, myoblasts were cultured for 5 days under PO_2_ 21% (red line) or 1% (blue line). **B** Immunolabelling of HIF1α (red). **C** Percentage of HIF1α-positive nuclei (**p < 0.01). **D** Percentage of EdU-positive or Ki67-positive nuclei (N.S.). **E** EdU incorporation (green). *Middle panel: Myocytes*. **F** 750,000 myoblasts were seeded per well. After 24h, myoblasts were switched to differentiation medium for 2 days under PO_2_ 21% (red line) or 1% (blue line). **G** Myogenin labelling (MGN, green IF). **H** Percentage of MGN-positive nuclei (*p < 0.05). *Lower panel: Myotubes*. **I** 750,000 myoblasts were seeded per well. After 24h, myoblasts were switched to differentiation medium for 4 days under PO_2_ 21% (red line) or 1% (blue line). **J** Myosin Heavy Chain (MyHC) immunolabelling (green IF). **K** Percentage of immunolabelled MyHC-positive area (N.S.) and Fusion Index quantification (*p < 0.05)

To evaluate whether HIF1α activation impacts the proliferation, differentiation, or fusion of healthy human myoblasts, we exposed LHCN-M2 myoblasts to hypoxic conditions known to induce HIF1α activation and nuclear translocation (Fig. 1A). Cells cultured at a standard PO_2_ of 21% (control condition of normoxia) presented a basal level of HIF1α activation as attested by immunofluorescence (IF) showing that 53% (± 6%) of their nuclei were HIF1α-positive (Fig. 1B, C). As expected, culture in hypoxic conditions (PO_2_: 1%) stabilized HIF1α with 90% (± 2%) of myoblasts with HIF1α-positive nuclei (Fig. 1B, C). Myoblast proliferation was then evaluated by EdU pulsing and revealed that approximately 15% of myoblasts had incorporated EdU under either normoxia or hypoxia (Fig. 1D, E). We also immunolabelled for the proliferation marker Ki67, and again, there was no statistical difference in the number of cells expressing Ki67 between normoxia or hypoxia.

Myogenic differentiation was assayed by IF to detect myogenin (MGN). Two days after induction of differentiation, 29% (± 3%) of myoblast nuclei were MGN-positive under normoxia, compared to 40% (± 2%) in hypoxia (Fig. 1F–H). Fusion index (FI) was then measured at day 4 of differentiation and revealed an increase of 31% under hypoxia as compared to normoxia. Myosin Heavy Chain (MyHC) is a late differentiation marker, and we found no difference in MyHC-positive area between normoxia and hypoxia (Fig. 1I–K). These results were confirmed in another healthy human muscle cell line (54-6) [35] (Fig. S1). In summary, hypoxia enhanced early myogenic differentiation and myocyte fusion into myotubes.

### Hypoxia increases early differentiation in a HIF1α-dependent manner but its effect on late differentiation is HIF1α-independent

To directly examine the contribution of HIF1α to the effects observed on human LHCN-M2 myoblasts during hypoxic conditions, gain and loss of function experiments were performed.

For HIF1α gain of function, we used cobalt chloride (CoCl_2_), a chemical prolyl hydroxylase inhibitor allowing sustained HIF1α activation under normoxia. First, a dose-response determined the optimal CoCl_2_ concentration that caused HIF1α stabilization in LHCN-M2 human myoblasts after 24h. 10 μM CoCl_2_ was the lowest dose allowing a significant increase of HIF1α-positive nuclei from 49% (± 6%) (basal level) to 96% (± 1%) (*p* < 0,01, one-way ANOVA) under normoxia. No statistical difference was observed regarding the number of HIF1α-positive nuclei between 10 μM CoCl_2_ and the higher doses tested (Fig. S2).

**Fig. 2:**
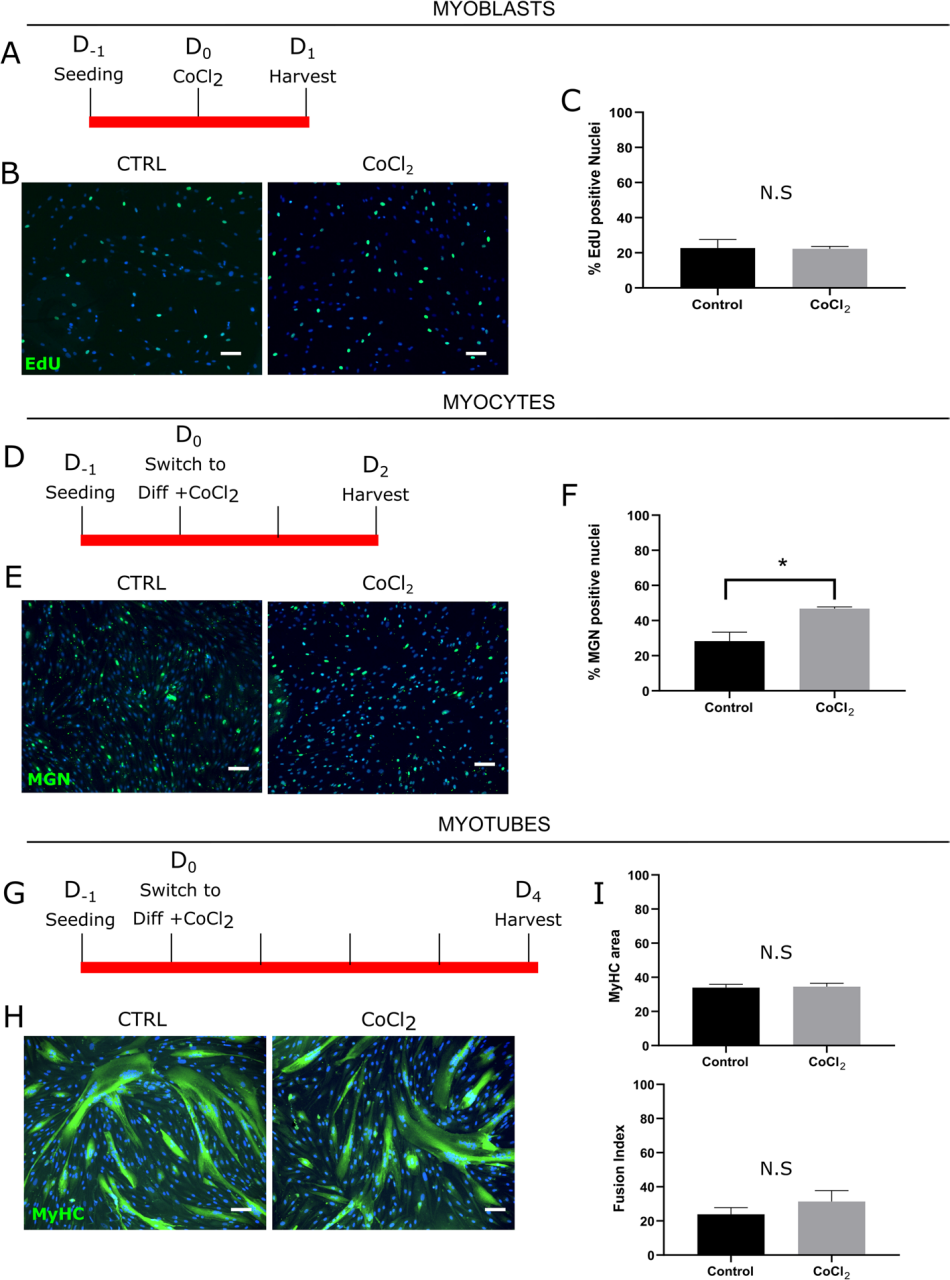
Gain-of-function experiments: a sustained HIF1α activation in normoxia increases early myoblast differentiation. LHCN-M2 myoblasts were seeded in a 6-well plate in standard conditions (PO_2_: 21%). After 24h, HIF1α activation was induced by treating cells with 10 μM CoCl_2_. After exposure, cells were fixed, proteins of interest were immunolabelled and positive nuclei were normalized to the total number of nuclei (DAPI; blue staining). Representative fields are shown. Scale bar: 100 μm. Experiments were performed on 3 independent cultures (each in triplicate) and mean ± SEM are represented and compared (*T*-test). *Upper panel: Myoblasts*. **A** 250,000 myoblasts were seeded per well and treated for 24h with 10 µM CoCl_2_. **B** EdU incorporation (green). **C** Percentage of EdU-positive cells (N.S.). *Middle panel: Myocytes*. **D** 750,000 myoblasts were seeded per well. After 24h, myoblasts were switched to differentiation medium with 10 µM CoCl_2_ for 2 days. **E** MGN labelling (green IF). **F** Percentage of MGN-positive nuclei (*p < 0.05). *Lower panel: Myotubes*. **G** 750,000 myoblasts were seeded per well. After 24h, myoblasts were switched to differentiation medium with 10 µM CoCl_2_ for 4 days. **H** MyHC labelling (green IF). **I** Percentage of immunolabelled MyHC-positive area (N.S.) and Fusion Index quantification (N.S.)

There was no significant difference in the proliferation rate between myoblasts cultured with 10 μM CoCl_2_, as compared to non-treated controls (Fig. 2A–C). However, we observed an increased percentage (47 ± 1%) of MGN-positive nuclei in myocytes treated with CoCl_2_ compared to the control group (28 ± 5%) (Fig. 2D–F). Interestingly, in contrast to myocytes exposed to hypoxia (Fig. 1K), CoCl_2_ treatment in normoxia did not have any effect on myocyte fusion into myotubes as attested by determining the fusion index. This suggested that the effects of hypoxia on late differentiation were independent of HIF1α. As per hypoxia, the immunolabelled MyHC-positive area also remained unchanged upon treatment with CoCl_2_ in normoxia (Fig. 2G–I).

**Fig. 3:**
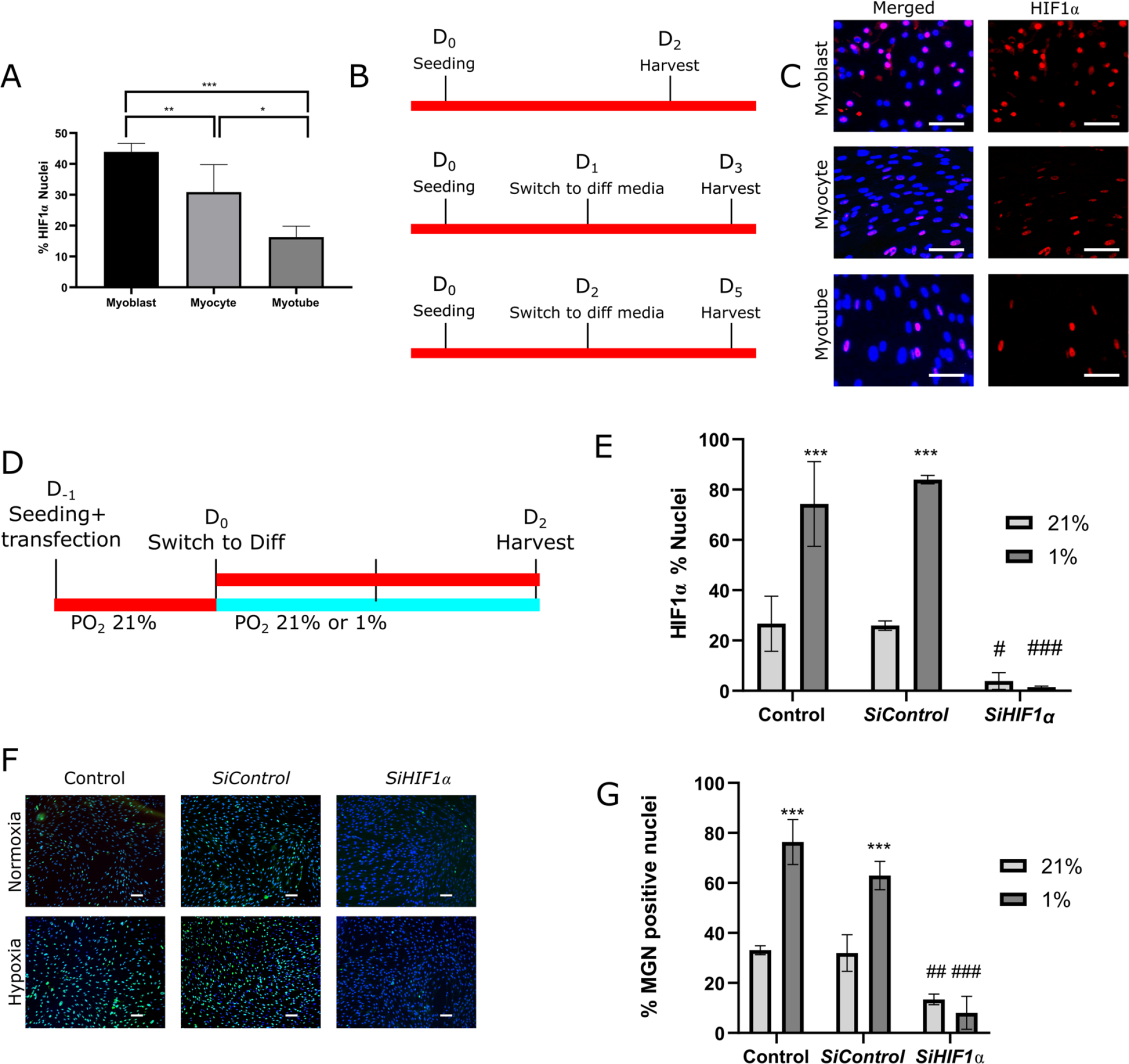
Loss-of-function experiments: HIF1α is critical for myogenic differentiation in both normoxia and hypoxia. **A**–**C** Basal level of HIF1α-positive nuclei in myoblasts, myocytes, and myotubes in normoxia. 750,000 LHCN-M2 myoblasts were seeded in a 6-well plate in standard conditions (PO_2_: 21%). After 24h, the myoblasts were switched to differentiation medium for 2 (myocytes) or 4 days (myotubes). **A** Percentage of HIF1α-positive nuclei normalized to the total number of nuclei (DAPI; blue). One-way ANOVA followed by Holm Sidak. *p < 0.05, **p < 0.01, ***p < 0.001. **B** Experiment timeline. **C** Representative images of HIF1α labelling (red IF). Scale bar: 100 μm. **D**, **E** Efficiency of HIF1α knockdown in myoblasts transfected with siRNAs directed against HIF1α (*siHIF1α*) as compared to control siRNA (*siControl*) and non-transfected cells (Control) in normoxia and hypoxia. 750,000 LHCN-M2 myoblasts were transfected with the indicated siRNA, then seeded in a 6-well plate in standard condition (PO_2_: 21%). After 24h, the myoblasts were switched to differentiation medium for 2 days either in hypoxia (PO_2_: 1%) or normoxia (PO_2_: 21%). **D** Experiment timeline. **E** Percentage of HIF1α-positive nuclei normalized to the total number of nuclei (DAPI; blue). Two-way ANOVA followed by Holm Sidak comparing 1% vs 21% (***p < 0,001); comparing *SiHIF1α* vs Control in normoxia (#*p *< 0,05); comparing *SiHIF1α* vs *SiControl* and vs Control in hypoxia (###*p *< 0,001). **F**, **G** Effect of HIF1α knockdown on myocytes in normoxia and hypoxia. 750,000 LHCN-M2 myoblasts were transfected and cultured as in **D** and **E**. **F** Representative images of MGN labelling (green IF) after transfection with siRNAs directed against HIF1α (*siHIF1α*) or *siControl*. Scale bar: 100 μm. **G** Percentage of MGN^+^ nuclei normalized to the total number of nuclei (DAPI; blue). Two-way ANOVA followed by Holm Sidak comparing 1% vs 21% (***p < 0,001); comparing *SiHIF1α* vs Control in normoxia (##*p *< 0,01); comparing *SiHIF1α* vs *SiControl* and vs Control in hypoxia (###*p *< 0,001). Experiments were performed on 3 independent cultures, each in triplicate

Since gain-of-function studies suggested that early differentiation of human myoblasts into myocytes was HIF1α-dependent, loss-of-function experiments were performed by using siRNAs against *HIF1α* mRNA (*SiHIF1α*). We first determined the percentage of HIF1α-positive nuclei in myoblasts, myocytes, and myotubes in normoxia (Fig. 3A–C). Interestingly, we noticed that the proportion of HIF1α-positive nuclei in myoblasts (45 ± 2%) decreased during the differentiation process, to reach only 16 ± 2% in myotubes. In myoblasts transfected with *SiHIF1α,* HIF1α knockdown was also confirmed by immunofluorescence. The quantification of HIF1α-positive nuclei in hypoxic conditions showed a 57-fold decrease in myoblasts transfected with *SiHIF1α* as compared to myoblasts transfected with the control siRNA (*SiControl*) (Fig. 3D, E and Fig. S3). As concerns the impact of HIF1α loss-of function on early differentiation, our results show a decreased percentage of MGN-positive nuclei in normoxia, indicating that a basal HIF1α level is critical for normal myogenic differentiation. As expected, the switch from normoxia to hypoxia caused a two-fold increase of MGN-positive nuclei in non-transfected myocytes, as well as in myoblasts transfected with the *SiControl*. This difference in the percentage of MGN-positive nuclei was no longer observed when myoblasts were transfected with *SiHIF1α* and differentiated into myocytes. There was no difference between the control (non-transfected cells) and the *SiControl* group, whatever the oxygen level in the ambient gas mixture (Fig. 3F, G). In summary, these data indicated that HIF1α was required for the precocious myogenic differentiation caused by hypoxia.

### DUX4 interferes with the normal function of HIF1α in muscle differentiation

Since our data highlighted a role of HIF1α in early myogenic differentiation and the HIF1α pathway was known to be perturbed in FSHD [16, 17, 28], we investigated whether DUX4 interfered with this process either in normoxic or hypoxic conditions.

**Fig. 4:**
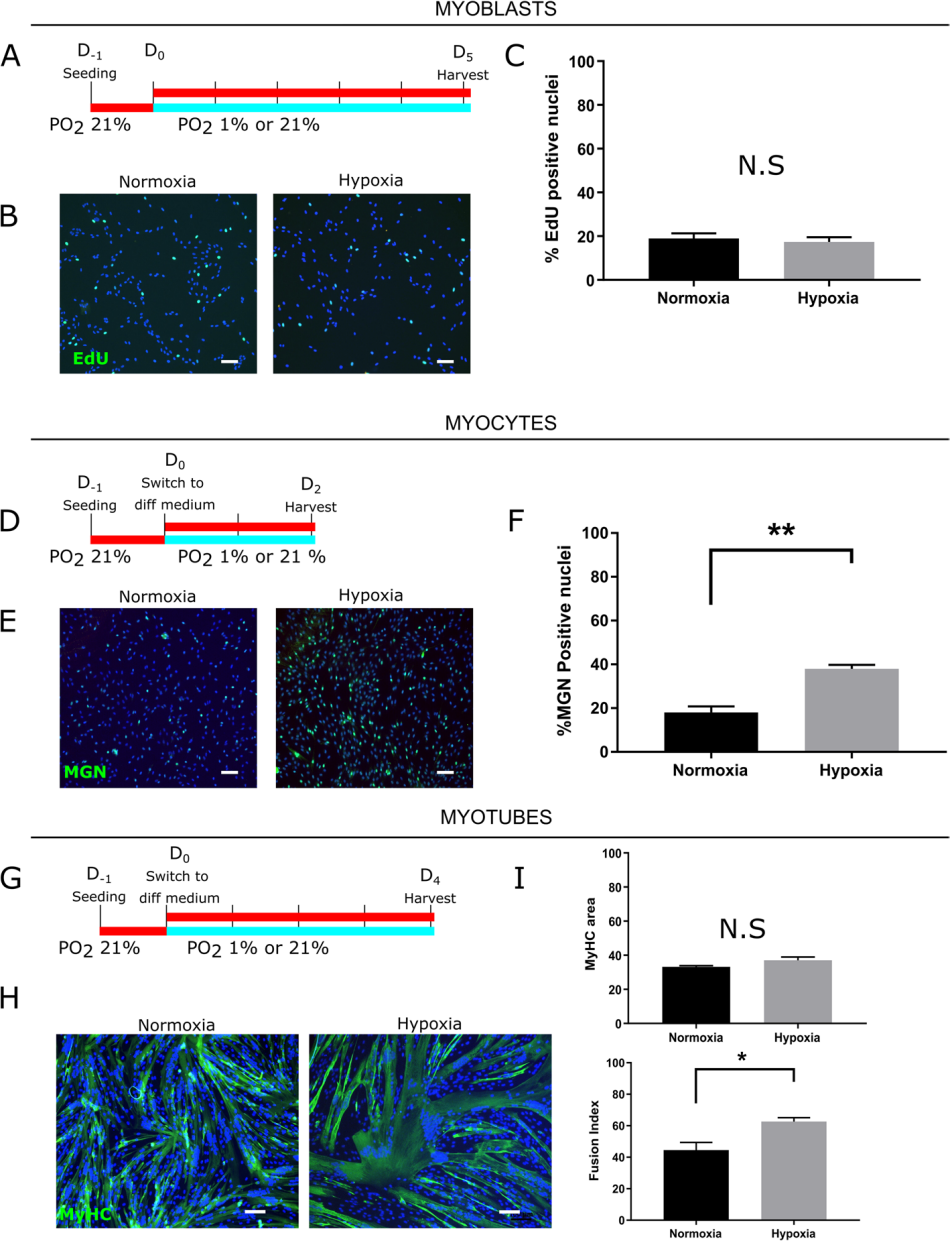
Hypoxia increases early differentiation and fusion of human LHCN-M2-iDUX4 myoblasts without DUX4 induction. LHCN-M2-iDUX4 myoblasts were seeded in a 6-well plate in standard conditions and 24h later exposed to hypoxia (PO_2_: 1%) or standard conditions (PO_2_: 21%). After exposure, cells were fixed, proteins of interest were immunolabelled and positive nuclei were normalized to the total number of nuclei (DAPI; blue staining). Representative fields are shown. Scale bar: 100 μm. Experiments were performed on 3 independent cultures (each in triplicate) and mean ± SEM are represented and compared (*T*-test). *Upper panel: Myoblasts*. **A** 250,000 uninduced LHCN-M2-iDUX4 myoblasts were seeded per well and cultured for 5 days under normoxia or hypoxia. **B** EdU incorporation (green). **C** Percentage of EdU-positive cells (N.S.). *Middle panel: Myocytes*. **D** 750,000 uninduced LHCN-M2-iDUX4 myoblasts were seeded per well. After 24h, myoblasts were switched to differentiation medium for 2 days under normoxia or hypoxia. **E** MGN labelling (green IF). **F** Percentage of MGN-positive nuclei (**p < 0.01). *Lower panel: Myotubes*. **G** 750,000 uninduced LHCN-M2-iDUX4 myoblasts were seeded per well. After 24h, myoblasts were switched to differentiation medium for 4 days under normoxia or hypoxia. **H** MyHC immunolabelling (green IF). **I** Percentage of immunolabelled MyHC-positive area (N.S.) and Fusion Index quantification (*p < 0.05)

We first assayed the impact of hypoxia-induced HIF1α activation on proliferation, differentiation, and fusion of LHCN-M2-iDUX4 myoblasts, engineered with a doxycycline (DOX)-inducible *DUX4* gene [36]. As expected, uninduced LHCN-M2-iDUX4 myoblasts behaved as per their parental LHCN-M2 myoblast line (Fig. 1): exposure to hypoxia did not modify the percentage of proliferating myoblasts that had incorporated EdU (Fig. 4A–C) but increased the percentage of MGN-positive nuclei (38 ± 2%) compared to normoxia (18 ± 3%) (Fig. 4D–F). There was no significant modification of the MyHC immunolabelled area upon hypoxia exposure, but LHCN-M2-iDUX4 myoblasts differentiated for 4 days had a higher fusion index (63 ± 3%) in hypoxia compared to normoxia (45 ± 5%) (Fig. 4G–I).

We also assessed the influence of culture conditions (Fig. S4 and Table S1). A medium enriched with growth factors and dexamethasone (PromoCell - Skeletal Muscle Cell Growth Medium) sometimes used by other groups [28, 37] increased (I) the basal doubling time of myoblasts (Fig. S4A and B) and (II) the proliferation rate upon hypoxia (Fig. S4C-D) compared to our standard medium (Table S1). However, hypoxia increased early differentiation (Fig. S4E-G) and fusion of myoblasts, but without changes in the MyHC-positive area (Fig. S4H-J) in either differentiation medium, although neither contained Dex (Table S1).

**Fig. 5:**
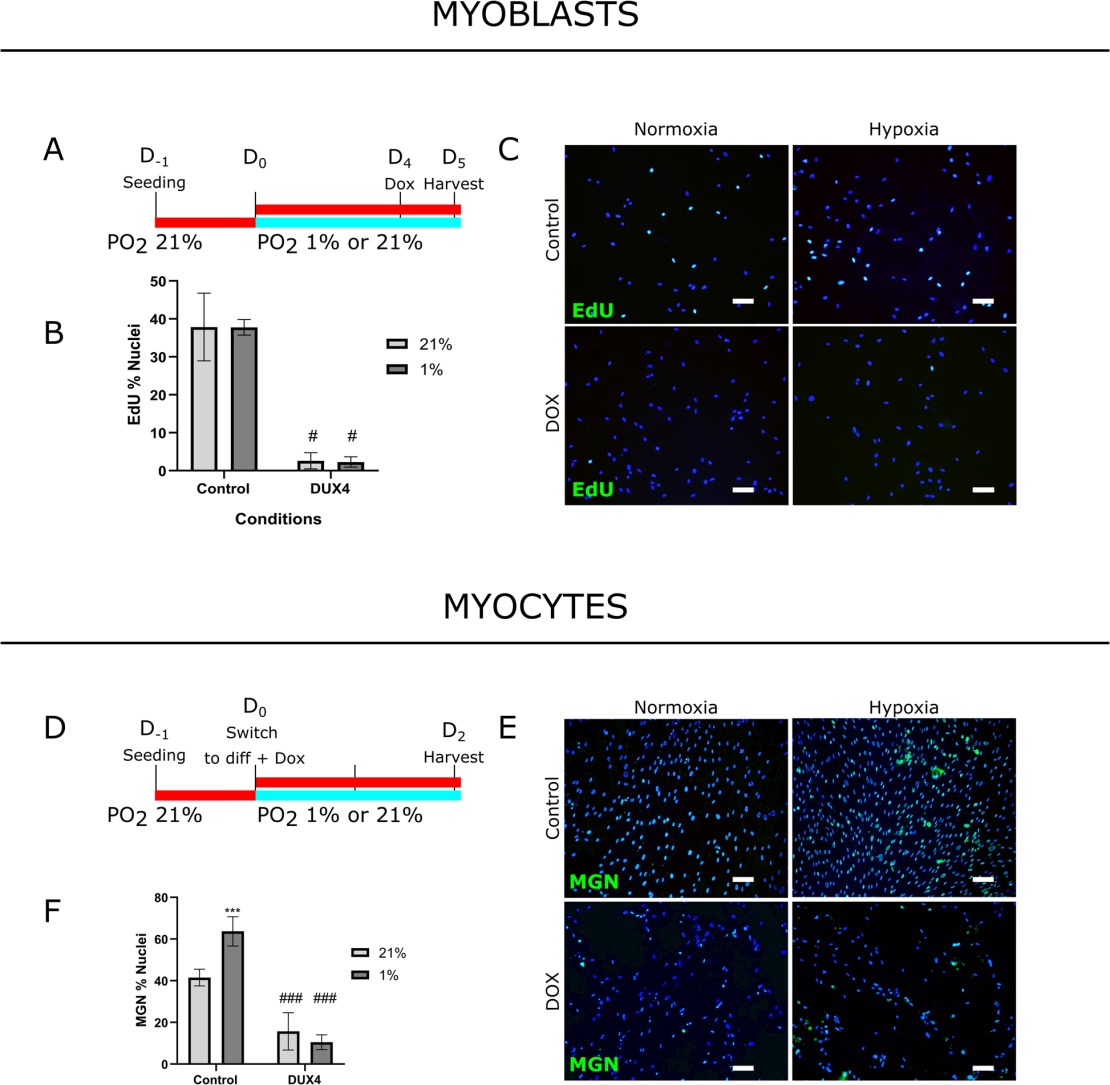
DUX4 interferes with the normal function of HIF1α during muscle differentiation. LHCN-M2-iDUX4 myoblasts were seeded in a 6-well plate. Myoblasts were then treated with doxycycline (62.5 ng/ml DOX) to induce DUX4 expression and exposed to hypoxia (PO_2_: 1%) or maintained in normoxia (PO_2_: 21%). After exposure, cells were fixed, proteins of interest were immunolabelled and positive nuclei were normalized to the total number of nuclei (DAPI; blue staining). Representative fields are shown. Scale bar: 100 μm. Experiments were performed on 3 independent cultures (each in triplicate) and mean ± SEM are represented and compared (Two-way ANOVA followed by Holm Sidak).* Upper panel: Myoblasts*. **A** 500,000 LHCN-M2-iDUX4 myoblasts were seeded per well and maintained in either normoxia or switched to hypoxic conditions for 4 days and then induced with DOX for 24h. **B** Percentage of EdU-positive cells. Two-way ANOVA followed by Holm Sidak. #*p* < 0.05, Control (uninduced) *vs* DUX4 (induced) in normoxia/hypoxia. **C** EdU incorporation (green). *Lower panel: Myocytes*. **D** 750,000 LHCN-M2-iDUX4 myoblasts were seeded per well. After 24h, myoblasts were switched to a differentiation medium and induced with DOX for 2 days under normoxia or hypoxia. **E** MGN immunolabelling (green IF). **F** Percentage of MGN-positive nuclei. Two-way ANOVA followed by Holm Sidak. ^###^*p* < 0.001, Control *vs* DUX4 in normoxia/hypoxia; ***p < 0.001, PO_2_ 1% *vs* 21%

We then investigated how DUX4 could influence HIF1α normal function in the myogenic program by using LHCN-M2-iDUX4 myoblasts and myocytes induced with 62.5 ng/ml of DOX for 24h or 48h, both in normoxia and hypoxia. DUX4 expression was checked using DUX4 immunolabelling (data not shown). In the myoblast stage, we observed a decreased percentage of EdU-positive nuclei upon DUX4 expression in normoxia (3 ± 1%) and hypoxia (2 ± 1%) conditions compared to the control without DOX induction (38 ± 5% EdU-positive nuclei in normoxia and 38 ± 1% in hypoxia) (Fig. 5A–C). As previously shown, in the absence of DUX4, no change was observed in myoblasts cultured in hypoxia compared to normoxia. In the myocyte stage (Fig. 5D–F), as expected, we observed a decreased percentage (16 ± 5%) of MGN-positive nuclei upon DUX4 expression in normoxia compared to the control without DOX induction (42 ± 2%). In the absence of DUX4, an increased percentage of MGN-positive nuclei was observed in myocytes cultured in hypoxia for the 2 days of differentiation (64 ± 4%) compared to normoxia (42 ± 3%) (Fig. 5F). In contrast, DUX4 caused a 6-fold decrease in the number of MGN-positive nuclei in myocytes exposed to hypoxia compared to myocytes in normoxia (Fig. 5D–F). In summary, our data indicated that DUX4 could interfere with the induction of early myogenic differentiation in hypoxia, an effect previously shown to be dependent on HIF1α.

## Discussion

Skeletal muscle regeneration plays a major role in the restoration of muscle homeostasis after injury or in the context of pathologies characterized by progressive muscle weakness and degeneration. This process is finely controlled by multiple cellular and molecular events [3].

Modulation of the oxygen levels can alter the regenerative capacity of SC in vitro [38]. Surprisingly, only few studies focused on the effect of hypoxia on human myogenesis [4]. Our data revealed that hypoxia had no effect on human myoblast proliferation but increased the percentage of MGN-positive myocytes as well as the fusion index during their differentiation into multinucleated myotubes. This is similar to the effect of hypoxia on bovine SC, where hypoxia enhanced myotube formation and stimulated the expression of *Myod*, *Myogenin*, and *Myhc* [39]. On the other hand, 5% PO_2_ inhibited differentiation and induced atrophy in murine C2C12 skeletal muscle cells, while 10 or 15% PO_2_ induced the formation of hypertrophic C2C12 myotubes [8]. Exposure of mouse myoblasts to hypoxia (PO_2_: 1%) strongly inhibited multinucleated myotube formation and expression of differentiation markers such as *Myogenin*, *Myod*, and *Myf5* [40]. In contrast though, C2C12 myoblasts preconditioned to hypoxia could form hypertrophic myotubes when differentiated under normoxia [6].

Several experimental parameters therefore influence the differentiation of myoblasts under hypoxia: duration, O_2_ tension, and time course, particularly the stage of differentiation when hypoxia is applied. Cell origin also appears to constitute a critical factor as low PO_2_ had deleterious effects on mouse myoblast differentiation while it enhanced this process in human and bovine myoblasts. It is also important to underline that cell cultures are usually performed in a humidified 95% air atmosphere, supplemented by 5% CO_2_, providing about 20% O_2_. In such a hyperoxic environment, cells reset their normoxic set-point [41]. Those conditions, commonly considered “normoxia,” are not representative of O_2_ partial pressure in tissues in vivo. In skeletal muscle, the physiological range of O_2_ level (termed “physioxia”) is significantly lower, about 4% O_2_ [42–45]. The interpretation and translation to whole organisms of data obtained in vitro have to be made with caution and take into account that cellular and molecular reactions to hypoxia may differ from those occurring in vivo.

We also highlighted the influence of the culture medium as we observed a positive effect of hypoxia on proliferation in a medium containing dexamethasone (Dex). As a potential limitation of the study, our experiments in the Dex-containing medium were only performed in uninduced LHCN-M2-iDUX4, since no influence of the DUX4-inducible cassette was shown upon hypoxia, as compared to the parental LHCN-M2 line. The synthetic glucocorticoid Dex has often been used due to its effects on muscle in vivo and on muscle cell cultures in vitro. Indeed, in mice with muscle injury, Dex significantly promotes muscle regeneration via a modulation of kinesin-1-motor activity required for the expression of particular MyHC isoforms, myoblast fusion, and myotube formation [46]. Moreover, a differentiation medium supplemented with Dex enhances the differentiation of C2C12 myoblasts when exposed to hypoxia, notably by increasing myotube length [47]. Interestingly, a functional separation was reported between HIF1α-mediated hypoxic response and mechanisms underlying glucocorticoid response regulation in human primary myoblasts. Indeed, Dex did not modify HIF1α expression or protein level and did not alter the expression of *PDK1*, a direct HIF1α target gene under either normoxic or hypoxic conditions [48]. However, Dex downregulated *VEGF* expression. Considering the effects of Dex, especially on muscle differentiation, and its inhibitory role on *DUX4* expression [49], we avoided its use.

If HIF1α is known as the principal effector of the hypoxic response, its role in skeletal muscle regeneration was only recently described, mainly using murine in vitro models. As a regulator of both embryonic and post-natal myogenesis, HIF1α is notably critical for the maintenance of SC quiescence in their hypoxic stem niche [11]. To determine the implication of HIF1α on hypoxia-mediated changes in human muscle cell differentiation, we performed gain and loss of function experiments by using CoCl_2_ and *siRNA* against *HIF1α*, respectively. As observed in hypoxia, we found no difference in the proliferation rate between myoblasts cultured with CoCl_2_, as compared to control myoblasts. However, an increased number of MGN-positive nuclei in myocytes treated with CoCl_2_ was observed, indicating precocious myogenic differentiation. This increase was no longer observed in loss of function experiments, when myocytes were transfected with *siRNA* targeting *HIF1α mRNA*. Our results indicate an HIF1α-dependent induction of early differentiation upon hypoxia. This phenomenon could involve the non-canonical WNT pathway which is one of the major myogenesis regulators [6]. Moreover, hypoxia controls SC identity and progression in the myogenic lineage by regulating gene expression through the HIF1α-WNT axis [50]. Therefore, reduced HIF1α expression or activity could prevent WNT pathway activation and therefore disturb myogenesis. As we have demonstrated in normoxic conditions, reduced MGN-positive nuclei in myocytes treated with *SiHIF1α* suggests that HIF1α is an important factor for early differentiation in a non-hypoxic context. Accordingly, *Hif1α* silencing in C2C12 muscle cells or its chemical inhibition by echinomycin significantly altered differentiation as shown by decreased *Myogenin* and *Myhc* expression [6]. Interestingly, adult mice with myoblast-specific *HIF1α/HIF2α* double KO presented normal muscle development as well as normal myofiber size and number suggesting that HIF1α and HIF2α were dispensable for normal muscle development. However, mice with postnatal SC-specific *HIF1α/2α* double KO had delayed injury-induced muscle repair notably associated with a reduced number of myoblasts during regeneration [11]. Finally, we found that CoCl_2_ did not have any effect on myocyte fusion into myotubes, suggesting that this process was HIF1α-independent. Similarly, another study reported that the effect of hypoxia on myogenesis was independent of HIF1α as ectopic expression of HIF1α using a retrovirus in C2C12 cells did not impact Myod protein level. Furthermore, hypertrophy of C2C12 myotubes occurs upon mild hypoxia (PO_2_: 10%) [8]. Although mechanisms underlying the effect of hypoxia on myoblast fusion remain poorly described, especially in human cells, the 10% PO_2_ increased the phosphorylation of mTOR and p70s6K, two important factors for protein synthesis and skeletal muscle hypertrophy [8].

HIF1α and the associated hypoxic response pathway are particularly of interest in hereditary muscular dystrophies because (I) a significant subgroup of patients present respiratory impairments and subsequent hypoxemia, and (II) a large group of muscular dystrophies are associated with regeneration defects and SC dysfunction [33, 34]. However, multifactorial pathological mechanisms can lead to an inadequate HIF1α activation in skeletal muscles of these patients [4]. In particular, the genetic defect per se is susceptible to disturb the hypoxic response pathway independently of hypoxemia. A better understanding of processes underlying hypoxia-associated effects on human muscle cell differentiation is necessary to delineate potential therapeutic targets to enhance muscle regeneration in pathological contexts. In FSHD, the causative agent DUX4 is responsible for HIF1α signaling disturbances [26]. The deregulated molecular network causing FSHD skeletal muscle dysfunction is still a major research topic. Meta-analyses highlighted the PAX7-HIF1α axis as critically disturbed in FSHD muscles [17]. Accordingly, the hypoxia response pathway was recently described as a key driver of DUX4-induced cell death in myoblasts [28]. The effect of DUX4 in hypoxic conditions is less known but mitochondrial dysfunction and oxidative stress are the main characteristics of FSHD muscle. As Heher et al. recently reported [51], DUX4 mediates oxidative metabolic impairments exacerbated in conditions of varying O_2_ tension.

Muscle differentiation and regeneration defects are well-known characteristics of FSHD [26]. The effect of DUX4 on myogenesis is largely described in the literature, particularly by Bosnakovski et al. [37] characterizing the LHCN-M2-iDUX4 cell model we used in our study. DUX4 is known to stop myoblast proliferation, to induce cell death [24, 52], to impair the myogenic program by reducing the expression of genes encoding myogenic factors [37] and by inducing a stem-cell-like transcriptional program [53], therefore leading to myoblast differentiation into hypotrophic myotubes [23]. Importantly, Heher et al. showed that hypoxia aggravates the hypotrophic FSHD myotube phenotype observed in normoxia in three independent patient-derived FSHD/control paired myoblast lines [51]. This is likely due to a metabolic misadaptation leading to exacerbated oxidative stress. However, the influence of DUX4 on HIF1α normal function in the context of muscle differentiation requires further investigation. Here, we report that DUX4 altered early differentiation of human muscle cells as shown by the decreased percentages of MGN-positive nuclei in the myocyte stage. Moreover, we found that DUX4 counteracted the hypoxia-mediated increase of MGN-positive nuclei in hypoxic culture conditions, suggesting that DUX4 interfered with the HIF1α role in early myogenic differentiation in a hypoxic environment. Accordingly, the powerful and negative effect of DUX4 on myogenesis has been underlined since even at a low level, DUX4 was able to deregulate myogenic gene expression [37].

**Fig. 6:**
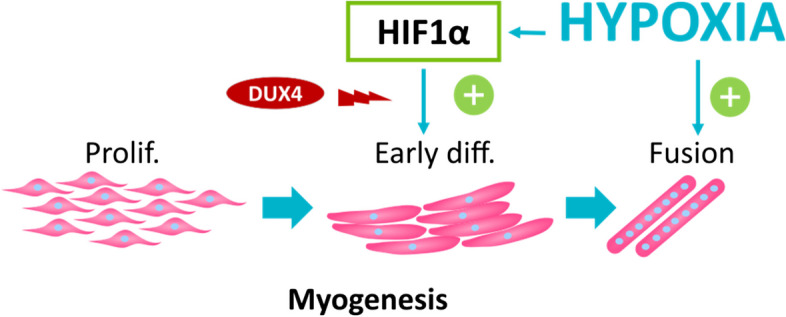
Summary diagram

In conclusion, our study highlights that hypoxia and sustained HIF1α activation do not alter the myoblast proliferation rate but increase their early differentiation. Hypoxia also enhances late differentiation, but this phenomenon is HIF1α-independent. We further discovered unexpected differences in DUX4/HIF1α interplay in proliferating or differentiating myoblasts in hypoxic conditions. Besides a role in which HIF1α can contribute to DUX4 toxicity in proliferating FSHD myoblasts [28], we also found that DUX4 suppressed the role of HIF1α in promoting early differentiation, thus interfering with FSHD muscle regeneration (Fig. 6). These opposite toxic/beneficial functions preclude the use of HIF1α inhibition as a simple therapeutic approach for FSHD.

## Material and methods

### Cell culture

Immortalized human myoblast lines LHCN-M2 and LHCN-M2-iDUX4 were kindly provided by Prof. M.Kyba (Lillehei Heart Institute, University of Minnesota, Minneapolis). The 54-6 cell line was kindly provided by Prof. V. Mouly (Myology Institute, Paris) and derived from the biceps of a mosaic FSHD1 patient; these cells harbor 13 *D4Z4* units in the FSHD locus and were used as healthy human myoblasts.

LHCN-M2 cells were cultured in a proliferation medium either composed of DMEM F12 (BioWest) supplemented with 20% FBS (Biowest) and 1% Penicillin/Streptomycin (P/S, Thermofisher) or with Skeletal Muscle Cell Growth Medium supplemented with 20% of FBS and the SupplementMix (PromoCell) (Table S1). 54-6 cells were cultured in DMEM high glucose (VWR) supplemented with 20% of FBS, 1% Ultroser (Pall Life Sciences), and 1% Penicillin/Streptomycin (P/S, Thermofisher). Cells were cultured at 37 °C under a 21% O_2_ and 5% CO_2_ atmosphere. For myogenic differentiation, cells were cultured on matrigel coated dishes (Corning) in proliferation medium until 100% confluence, before washing once with PBS and differentiated for 2 days (for myocytes) and 4 days (for myotubes) using differentiating medium (Table S1). LHCN-M2 cells were differentiated by medium switch to DMEM/F12 (Corning Cellgro), supplemented with human insulin 10 μg/ml (Sigma), bovine apo-transferrin 100 μg/ml (Sigma), and 1% Penicillin/Streptomycin (P/S, Thermofisher) or with differentiation medium supplemented with Differentiation Medium Supplement Mix (PromoCell). 54-6 cells were differentiated in DMEM/F12 (Corning Cellgro), supplemented with human insulin 10 μg/ml (Sigma), bovine apo-transferrin 100 μg/ml (Sigma), and 1% Penicillin/Streptomycin (P/S, Thermofisher).

Cell cultures under hypoxic conditions were performed in a separate incubator (Nuaire) at 37 °C in a 5% CO_2_ atmosphere with 1% O_2_. To keep O_2_ concentration constant, a gas mixture composed of 99% N_2_ and 1% O_2_ was injected into the incubator. For hypoxic cultures, all media were preconditioned through incubation of at least 24h under 5% CO_2_ atmosphere with 1% O_2_.

In experiments with HIF1α stabilization, cobalt chloride (CoCl_2_; Sigma) was used. Upon its dissociation, Co^2+^ ions will substitute Fe^2+^ ions present in PHD enzymes and inhibit their activity. Therefore, even in normoxia, HIF1α subunits are not hydroxylated nor degraded. The 10 mM CoCl_2_ stock solution was prepared in a culture medium, filtered and kept at −20°C. Finally, the solution was added to the medium at a final concentration of 10 μM.

For DUX4 induction, a 50 mg/ml doxycycline stock solution was prepared in sterile water and kept at -20°C. Then, a 50 μg/μl working solution was prepared by diluting the stock solution in sterile water and kept at 4 °C. LHCN-M2-iDUX4 cells were induced at the dose of 62.5ng/ml of doxycycline for 24 or 48h after seeding.

For proliferation assay, we used EdU (5-ethynyl-2 ´-deoxyuridine), a thymidine nucleoside analog incorporated into DNA during the S phase of the cell cycle. Cells were incubated with EdU reagent 2 h prior to fixation. EdU incorporation was performed using Click-iT™ EdU Cell Proliferation Kit for Imaging according to the manufacturer’s instructions (Invitrogen). We also detected Ki67, a protein marker of cell proliferation expressed during all active phases of the cell cycle (G1, S, G2, and M) but absent in G0; it was detected by immunofluorescence.

For *SiRNA* transfection, 4 *siRNAs* directed against the *HIF1α mRNA* were used (FlexiTube SI02664431, SI02664053, SI04262041, SI04361854; Qiagen) as well as “all star negative control” (Qiagen). Redundancy experiments using several distinct *siRNAs* targeting different sequences of the same mRNA prevent sequence-derived off-target effects [54, 55]. Per well, 10 nM of *SiRNA* was mixed with 100 μL Optimem (Gibco) and 1 μL of Lipofectamin RNAiMax (ThermoFisher). The concentration of each siRNA within the *siRNA* stock solution is therefore only 2.5 nM, further limiting any off-target effects. Following a 20-min incubation, the mixture was added to myoblasts resuspended in 500 μl of proliferation medium without Penicillin/Streptomycin in a 24-well plate. Twenty-four hours later, cells were washed with PBS and the differentiation medium was added to the culture.

### Immunofluorescence

Cells were fixed with 4% paraformaldehyde/PBS for 10 min and washed twice with PBS. Cells were permeabilized with 0.5% TritonX-100/PBS for 10 min and then incubated with a blocking solution (5% normal goat serum (Biowest), TritonX-100/PBS) for 1 h at room temperature. Cells were then incubated with primary antibodies (anti-HIF1α, rabbit monoclonal, ab179483, 1:500, Abcam, anti-myogenin, mouse monoclonal, F5D, DSHB, 1:10, anti-MyHC MF20, mouse monoclonal, DSHB, 1:100 and Ki67, mouse monoclonal, ab8191, Abcam) at 4 °C overnight. Cells were subsequently washed 3 times with PBS before being incubated with secondary antibodies Alexa 555 Goat anti-rabbit IgG (1:500, Biotium) and/or Alexa 488 Goat anti-mouse Ig (1:500, Biotium) at room temperature for 1 h. Cells were washed three times with PBS after incubation with the secondary antibody. Finally, immunolabelled cells were mounted with EverBrite Mounting Medium with DAPI (Biotium) for nuclear staining. Images were taken with a Nikon Eclipse 80i microscope and merged using NIS-Elements software. Quantification was performed by using Image J software. Six fields per well were quantified.

For proliferation and early differentiation, we counted EdU-positive, Ki67-positive, and MGN-positive nuclei, respectively and expressed as a percentage of the total number of nuclei in a field. For early differentiation, MGN immunolabelling was used since this myogenic regulatory factor is expressed at early stages of skeletal muscle cell differentiation [56]. For late differentiation, we used two different readouts: the area labelled for MyHC, a late differentiation marker detected by immunofluorescence [3], and the fusion index which corresponds to the number of nuclei inside myotubes (≥ 2 nuclei) as a percentage of the total number of nuclei in a field [57].

### Statistics

Normality tests (Shapiro-Wilk) were performed on each data set to assess data distribution and select the appropriate statistic test. Statistical analyses were done using GraphPad Prism software, version 8.02, and SigmaPlot software, version 14. Concerning Edu, MGN, MHC, and FI measures in hypoxia or with/without CoCl_2_ treatment, statistical analyses were performed using an unpaired *t*-test. Concerning experiments aiming to assess the effect of *SiRNA* or DOX induction in hypoxia or normoxia, data were analyzed by a two-way ANOVA followed by Holm-Sidak post hoc test. Differences were considered statistically significant at a *P*-value < 0.05. All data are represented as mean ± SEM.

### Supplementary Information

**Additional file 1: Figure S1.** Hypoxia enhances early and late differentiation of control 54-6 human myoblasts.

**Additional file 2: Figure S2.** Effect of treatment with Cobalt Chloride (CoCl_2_) on human LHCN-M2 myoblasts: dose response experiment.

**Additional file 3: Figure S3.** Validation of HIF1α loss of function upon siRNA use.

**Additional file 4: Figure S4.** Hypoxia enhanced human LHCN-M2-iDUX4myoblast proliferation when cultured in Dexamethasone-enriched PromoCell-Skeletal Muscle Cell Growth Medium.

**Additional file 5: Table S1.** Composition of culture media. 

## Data Availability

All data supporting the findings of this study are available within the article and its Supplementary Information.
