# Supplementary material for: Hypoxia enhances human myoblast differentiation: involvement of HIF1α and impact of DUX4, the FSHD causal gene
Source: Skelet Muscle. 2023 Dec 16;13:21. doi: 10.1186/s13395-023-00330-2 (PMC10724930; doi:10.1186/s13395-023-00330-2)
Supplement: Supplementary file 1 — Additional file 1: Figure S1. Hypoxia enhances early and late differentiation of control 54-6 human myoblasts. [file 13395_2023_330_MOESM1_ESM.pdf]

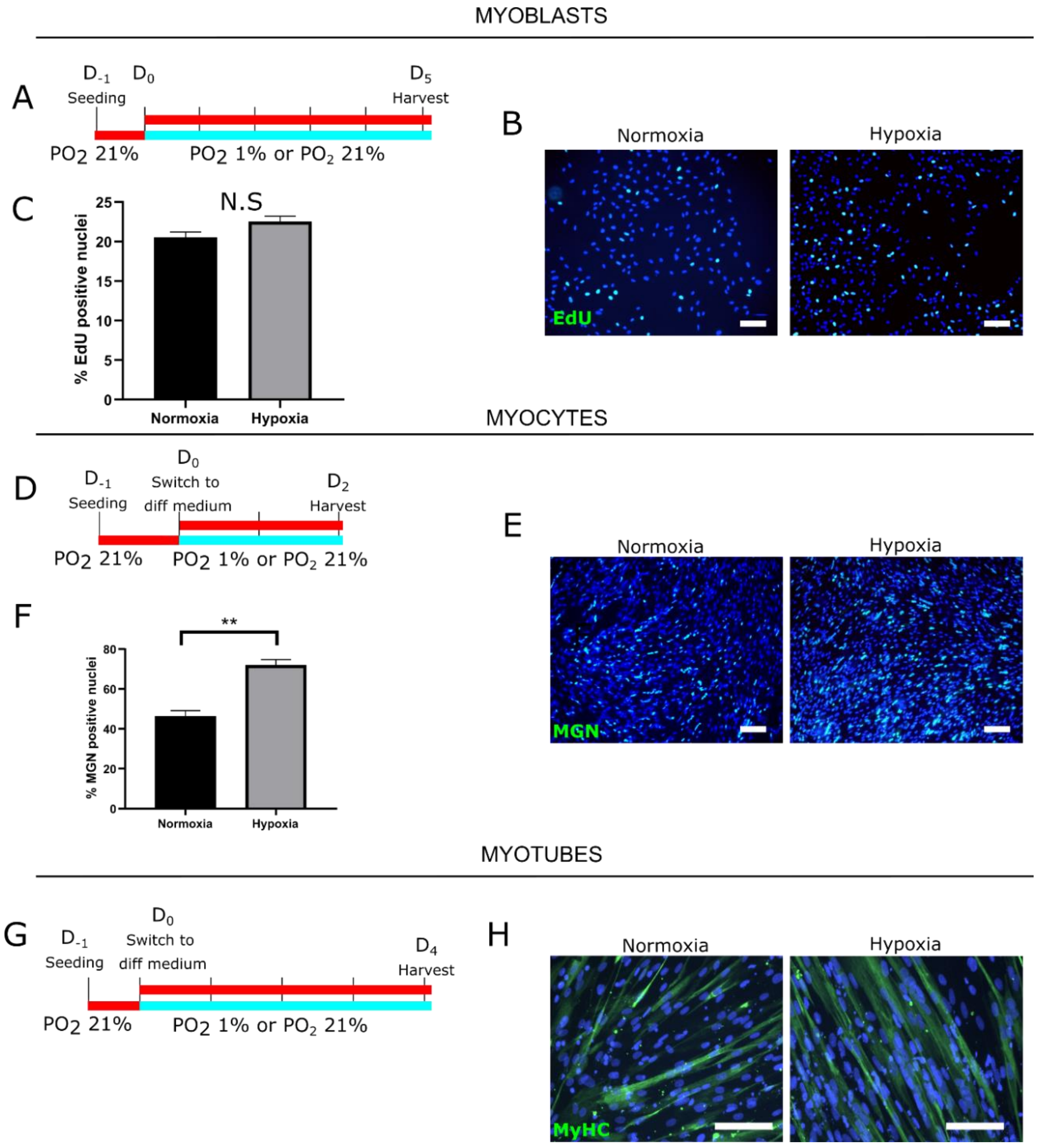

Fig. S1. Hypoxia enhances early and late differentiation of control 54-6 human myoblasts. 54-6 myoblasts were seeded in a 6-well plate in standard conditions and 24h later exposed to hypoxia (PO_2: 1% - blue line) or maintained in standard conditions (PO_2 21% - red line). After exposure, cells were fixed, proteins of interest were immunolabelled and positive nuclei normalized to the total number of nuclei (DAPI; blue staining). Representative fields are shown (Scale bar: 100 µm). Experiments were performed on 2 (myoblasts) or 3 (myocytes) independent cultures (each in triplicate) and mean ± SEM are represented and compared (*T*-test). *Upper panel: Myoblasts.* **A**. 250,000 54-6 myoblasts were seeded per well. After 24h, myoblasts were cultured for 5 days under PO_2 21% (red line) or 1% (blue line). **B.** EdU incorporation (green). **C.** Percentage of EdU-positive nuclei. *Middle panel: Myocytes.* **D.** 750,000 54-6 myoblasts were seeded per well. After 24h, myoblasts were switched to differentiation medium for 2 days under PO_2 21% (red line) or 1% (blue line). **E**. Myogenin labelling (MGN, green IF). **F**. Percentage of MGN-positive nuclei (**p<0.01). *Lower panel: Myotubes.* **G.** 750,000 54-6 myoblasts were seeded per well. After 24h, myoblasts were switched to differentiation medium for 4 days under PO_2 21% (red line) or 1% (blue line). **H.** Myosin Heavy Chain (MyHC) immunolabelling (green IF).
