# Supplementary material for: Hypoxia enhances human myoblast differentiation: involvement of HIF1α and impact of DUX4, the FSHD causal gene
Source: Skelet Muscle. 2023 Dec 16;13:21. doi: 10.1186/s13395-023-00330-2 (PMC10724930; doi:10.1186/s13395-023-00330-2)
Supplement: Supplementary file 2 — Additional file 2: Figure S2. Effect of treatment with Cobalt Chloride (CoCl2) on human LHCN-M2 myoblasts: dose response experiment. [file 13395_2023_330_MOESM2_ESM.pdf]

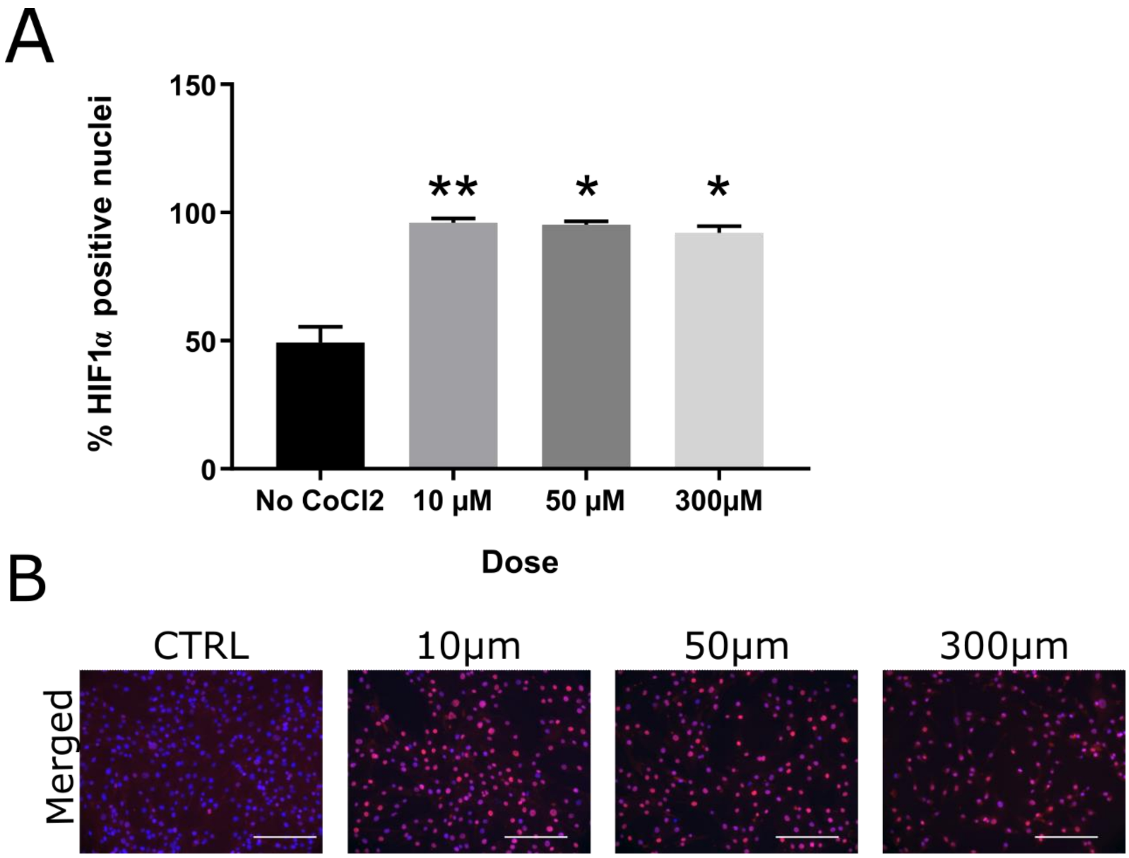

Fig. S2. Effect of treatment with Cobalt Chloride ($CoCl_2$) in LHCN-M2 myoblasts: dose-response experiment. **A.** Percentage of HIF1α-positive nuclei (red IF) normalized to the total number of nuclei (DAPI; blue staining). Experiments were performed on 3 independent cultures (each in triplicate) and mean ± SEM are represented and compared. One-way ANOVA followed by Holm-Sidak, *$p<0.05$, **$p<0.01$ **B.** Representative fields: HIF1α immunolabelling (red IF). Scale bar: 100 µm.
