# Supplementary material for: Hypoxia enhances human myoblast differentiation: involvement of HIF1α and impact of DUX4, the FSHD causal gene
Source: Skelet Muscle. 2023 Dec 16;13:21. doi: 10.1186/s13395-023-00330-2 (PMC10724930; doi:10.1186/s13395-023-00330-2)
Supplement: Supplementary file 3 — Additional file 3: Figure S3. Validation of HIF1α loss of function upon siRNA use. [file 13395_2023_330_MOESM3_ESM.pdf]

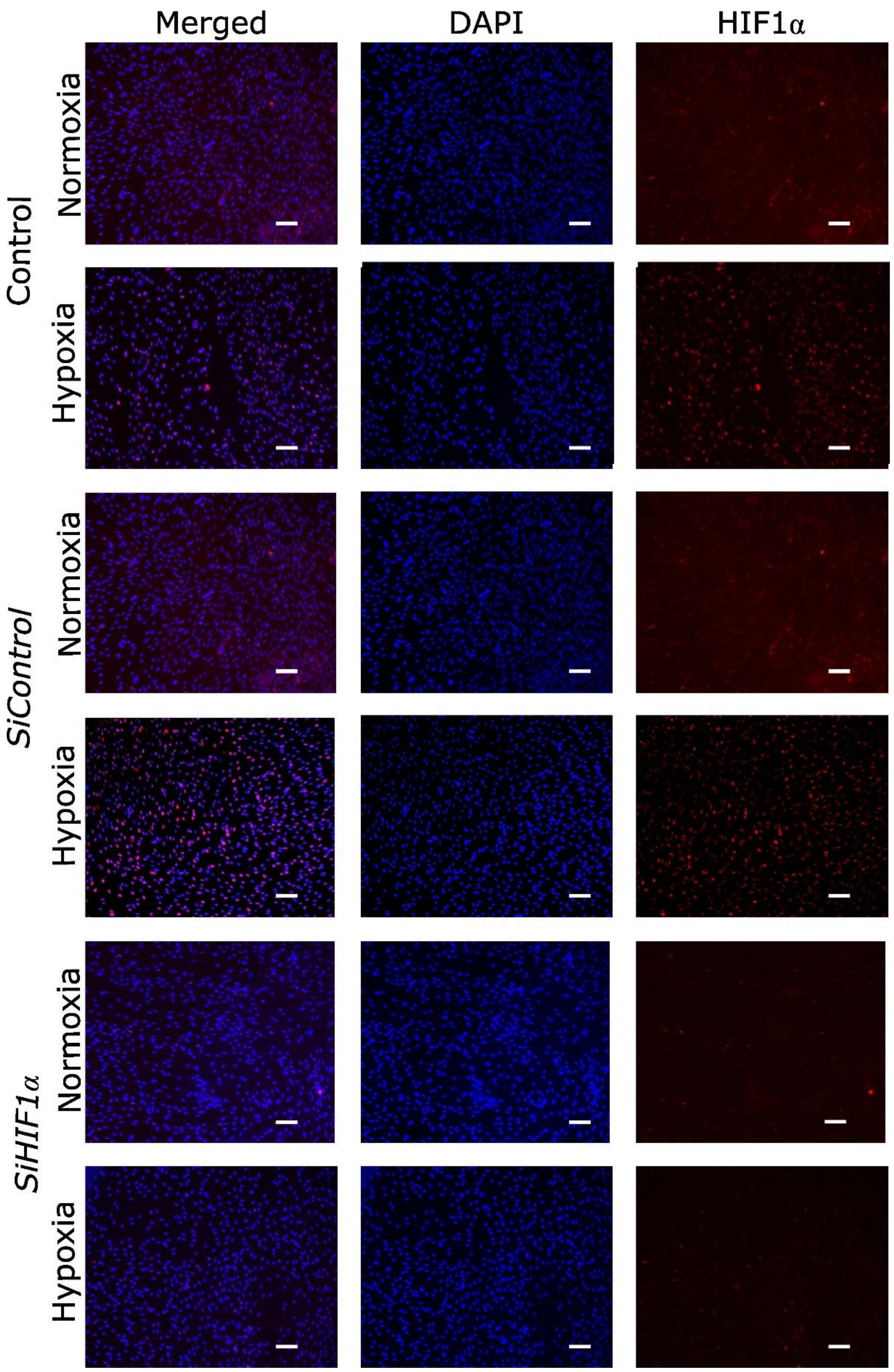

Fig. S3. Validation of HIF1α loss of function upon siRNA use. Representative fields: HIF1α immunolabelling (red IF). Nuclei are stained using DAPI (blue). Scale bar: 100μm. Experiments were performed on 3 independent cultures, each in triplicate.
