# Supplementary material for: Hypoxia enhances human myoblast differentiation: involvement of HIF1α and impact of DUX4, the FSHD causal gene
Source: Skelet Muscle. 2023 Dec 16;13:21. doi: 10.1186/s13395-023-00330-2 (PMC10724930; doi:10.1186/s13395-023-00330-2)
Supplement: Supplementary file 4 — Additional file 4: Figure S4. Hypoxia enhanced human LHCN-M2-iDUX4myoblast proliferation when cultured in Dexamethasone-enriched PromoCell-Skeletal Muscle Cell Growth Medium. [file 13395_2023_330_MOESM4_ESM.pdf]

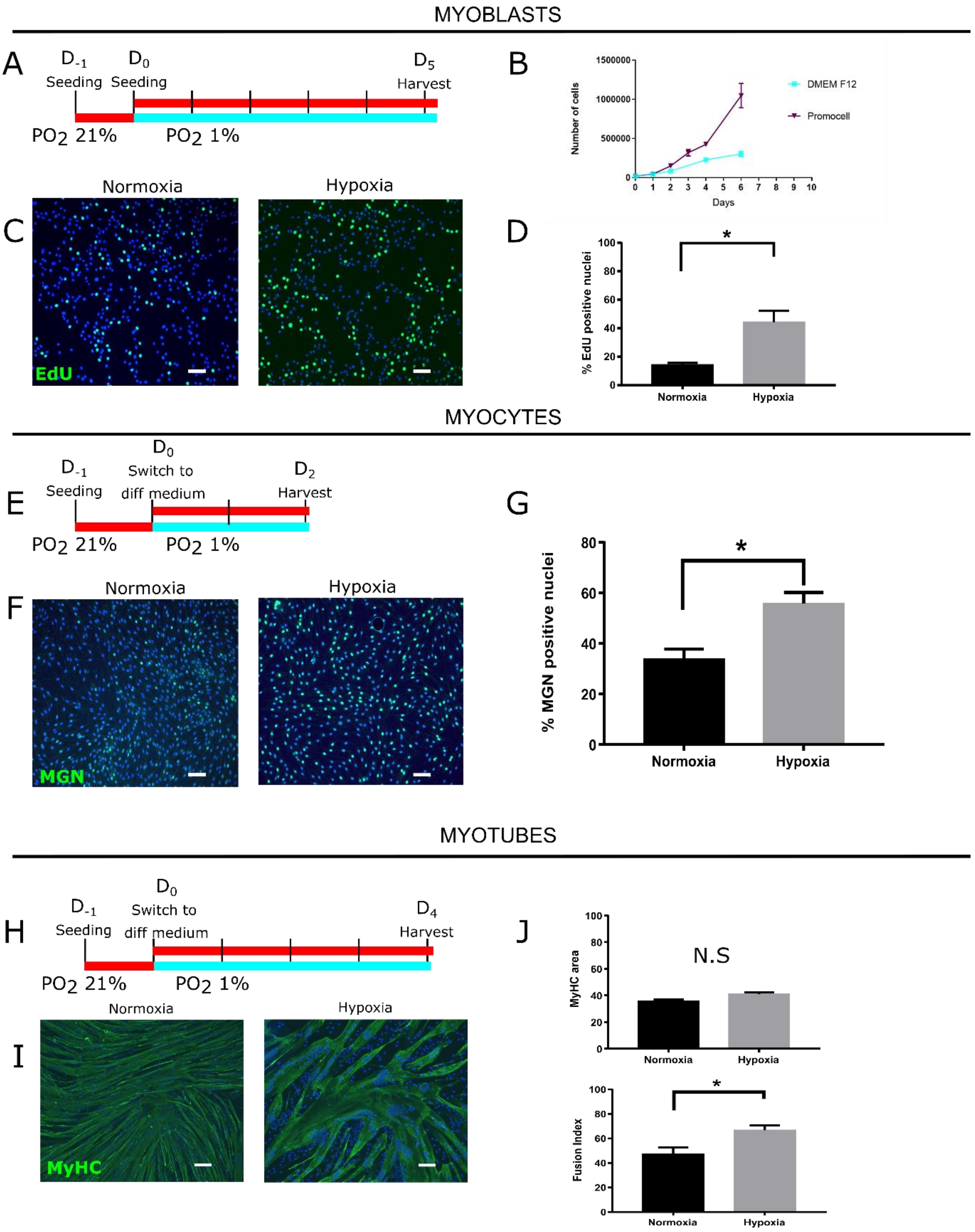


**Fig. S4.** Hypoxia enhanced human LHCN-M2-iDUX4 myoblast proliferation when cultured in Dexamethasone-enriched PromoCell-Skeletal Muscle Cell Growth Medium. PromoCell-Skeletal Muscle Cell Growth Medium, a ready-to-use medium specially designed for muscle cell culture was compared to a classical DMEM F12 formulation. In contrast to DMEM F12, the PromoCell proliferation medium is growth factor-enriched and contains dexamethasone (Dex) (**Table S1**), a glucocorticoid often used to stimulate myoblast proliferation and promote cell growth. This is important since Dex influences HIF1 $\alpha$ pathway components. LHCN-M2-iDUX4 myoblasts were seeded in a 6-well plate in standard atmosphere conditions and 24h later exposed to hypoxia

($PO_2$: 1% - blue line) or maintained in standard conditions ($PO_2$: 21% - red line). After exposure, cells were fixed, proteins of interest were immunolabelled (green IF) and positive nuclei were normalized to the total number of nuclei (DAPI; blue staining). Representative fields are shown. Scale bar: 100 µm. Experiments were performed on 3 independent cultures (each in triplicate) and mean ± SEM are represented and compared (*T*-test). *Upper panel: Myoblasts.* Myoblasts cultured in PromoCell-Skeletal Muscle Cell Growth Medium proliferated twice as fast as cells cultured in DMEM F12 (doubling time of 24.2h and 40.5h in PromoCell and DMEM F12, respectively. **A**. 250,000 myoblasts were seeded per well. After 24h, myoblasts were cultured for 5 days in the PromoCell proliferation medium under $PO_2$ 21% (red line) or 1% (blue line). **B.** Effect of DMEM F12 and PromoCell proliferation media on LHCN-M2-iDUX4 proliferation baseline. Growth curve. **C.** EdU incorporation (green). **D.** Percentage of EdU-positive cells (*$p<0.05$). Hypoxia doubled the percentage of EdU-positive cells. *Middle panel: Myocytes.* There remained an increased percentage of MGN-positive nuclei in myocytes cultured in the PromoCell medium under hypoxia during the 2 days of differentiation compared to normoxia. **E.** 750,000 myoblasts were seeded per well. After 24h, myoblasts were switched to Skeletal Muscle Differentiation Medium (PromoCell) for 2 days under $PO_2$ 21% (red line) or 1% (blue line). **F**. MGN immunostaining (green IF). **G**. Percentage of MGN-positive nuclei (*$p<0.05$). *Lower panel: Myotubes.* Hypoxia in Skeletal Muscle Differentiation Medium (PromoCell) had no significant effect on myotube MyHC-positive area but increased fusion index compared to normoxia. **H.** 750,000 myoblasts were seeded per well. After 24h, myoblasts were switched to Skeletal Muscle Differentiation Medium (PromoCell) for 4 days under $PO_2$ 21% (red line) or 1% (blue line). **I.** MyHC immunolabelling (green IF). **J.** Percentage of immunolabelled MyHC-positive area (N.S.) and fusion index quantification (*$p<0.05$). Thus growth factor-enriched culture medium containing Dex influenced the effect of hypoxia on myoblast proliferation. Upon differentiation, hypoxia increased early differentiation and myocyte fusion whatever the culture medium. Regarding this point, it is important to note that in contrast to the PromoCell proliferation medium, the PromoCell differentiation medium did not contain Dex (**Table S1**), even if myoblasts were submitted to its effect during the proliferation phase.
