# Supplementary material for: Hypoxia enhances human myoblast differentiation: involvement of HIF1α and impact of DUX4, the FSHD causal gene
Source: Skelet Muscle. 2023 Dec 16;13:21. doi: 10.1186/s13395-023-00330-2 (PMC10724930; doi:10.1186/s13395-023-00330-2)
Supplement: Supplementary file 5 — Additional file 5: Table S1. Composition of culture media. [file 13395_2023_330_MOESM5_ESM.pdf]

Table S1. Composition of culture media

**PromoCell proliferation medium**	**DMEM F12 proliferation medium**
Skeletal Muscle Cell Growth Medium (PromoCell)	DMEM -Ham's F12 medium (Biowest)
20% FBS	20% FBS
1% Penicillin Streptomycin	1% Penicillin Streptomycin
Fetuin (bovine) 50 µg / ml	
Epidermal Growth Factor (recombinant human) 10 ng / ml	
Fetal Calf Serum 0.05 ml / ml	
Basic Fibroblast Growth Factor (recombinant human) 1 ng / ml	
Insulin (recombinant human) 10 µg / ml	
Dexamethasone 0.4 µg / ml	
**PromoCell Differentiation Medium**	**DMEM F12 differentiation medium**
Skeletal Muscle Differentiation Medium (PromoCell)	DMEM -Ham's F12 medium (Biowest)
1% Penicillin Streptomycin	1% Penicillin Streptomycin
Human insulin 10 µg/ml	Bovine apo-transferrin 100 µg/ml
	Human insulin 10 µg/ml
